# Supplementary material for: Seroprevalence Assessment of Anti-Varicella Antibodies among Adults in the Province of Florence (Italy)
Source: Vaccines (Basel). 2024 Sep 16;12(9):1056. doi: 10.3390/vaccines12091056 (PMC11435818; doi:10.3390/vaccines12091056)
Supplement: Supplementary file 1 [file vaccines-12-01056-s001.zip › vaccines-3146319-supplementary.pdf]

Supplementary tables

Supplementary table 1. Differences based on sex for serological status by age group

		Sex (N)		Fisher's exact Test	
**Age groups**	**Serological status**	**Female**	**Male**	**Value**	***p***
18-29	negative	5	4	0.0936	1.000
	positive	24	24		
30-39	negative	4	4	0.0022	1.000
	positive	29	28		
40-49	negative	4	4	0.0503	1.000
	positive	39	33		
50-64	negative	3	2	0.1488	1.000
	positive	45	43		
>64	negative	6	3	0.3423	0.732
	positive	68	52		
Total	negative	22	17	0.1425	0.739
	positive	205	180		

Supplementary table 2. Differences based on nationality for serological status by age group

		**Nationality (N)**		**Fisher's exact Test**	
**Age groups**	**Serological status**	**Italian**	**Non-Italian**	**Value**	***p***
18-29	negative	6	3	2.4740	0.141
	positive	42	6		
30-39	negative	6	2	0.0647	1.000
	positive	45	12		
40-49	negative	6	2	0.1389	0.657
	positive	58	14		
50-64	negative	4	1	0.3384	0.475
	positive	78	10		
>64	negative	9	0	0.2304	1.000
	positive	117	3		
Total	negative	31	8	2.5213	0.126
	positive	340	45		
